# Association between atherosclerosis and Alzheimer's disease: A systematic review and meta‐analysis

**DOI:** 10.1002/brb3.1601

**Published:** 2020-03-11

**Authors:** Beijia Xie, Xinrui Shi, Yi Xing, Yi Tang

**Affiliations:** ^1^ Department of Neurology Innovation Center for Neurological Disorders Xuanwu Hospital National Clinical Research Center for Geriatric Diseases Capital Medical University Beijing China; ^2^ Beijing Key Laboratory of Geriatric Cognitive Disorders Neurodegenerative Laboratory of Ministry of Education of the People's Republic of China Beijing China

**Keywords:** Alzheimer's disease, atherosclerosis, carotid intima–media thickness, meta‐analysis

## Abstract

**Background:**

To evaluate the relationship between atherosclerosis and Alzheimer's disease (AD), we conducted a systematic review and meta‐analysis to study the difference of carotid intima–media thickness (CIMT) and the prevalence of atherosclerosis between AD patients and non‐AD controls.

**Methods:**

The studies on the association between atherosclerosis and AD were manually searched in PubMed, Embase, Cochrane Library, and CNKI (China National Knowledge Infrastructure) spanned to September 2018 according to PRISMA (the Preferred Reporting Items for Systematic Reviews and Meta‐Analyses) guidelines.

**Results:**

Thirteen studies were included in the final analysis, seven studies with data on the mean CIMT (610 cases and 417 controls) and ten studies reporting on the prevalence of atherosclerosis (1,698 cases and 6,452 controls). Compared with controls, AD group showed a significantly higher CIMT (overall standard mean difference = 0.94; 95% CI, 0.48–1.40; *p* < .0001) and an increased prevalence of atherosclerosis (OR = 1.46; 95% CI, 1.26–1.68; *p* < .0001).

**Conclusions:**

Atherosclerosis is significantly associated with AD. CIMT might be a useful marker to predict the risk of AD and assess the vascular burden. The finding is also important for possible prevention and treatment of AD in the future.

## Graphical abstract

Atherosclerosis is significantly associated with Alzheimer's disease (AD). Carotid intima–media thickness might be a useful marker to predict the risk of AD and assess the vascular burden. The finding is also important for possible prevention and treatment of AD in the future.

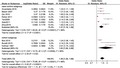

## 1 INTRODUCTION

Alzheimer's disease (AD) is a progressive neurodegenerative disorder characterized by amyloid‐β (Aβ) deposition, excessive phosphorylation of tau protein, and neurofibrillary tangles (Hardy & Selkoe, 2002; Prince et al., 2013). It has a long preclinical phase and causes impairment of cognitive, disability, and dependency of daily life (Moutinho & Landreth, 2017). AD is the most common type of dementia (Alzheimer's Association, 2016; Goodman et al., 2017) and accounts for about 60%–70% in dementia cases (World Health Organization, 2019). Under the background of an aging crisis (Ortman, Velkoff, & Hogan, 2014), AD seriously threatens the health of older people and results in enormous public burdens and family stress (Alzheimer’s Association, 2015; Jia et al., 2018; Wimo et al., 2017). Data showed that there are about 50 million people around the world who have dementia, and approximately 82 million people will have dementia in 2030 (World Health Organization, 2019). It was estimated that the global cost for dementia was about US $818 billion in 2015 (Wimo et al., 2017). Without effective treatment, it remains vital for explorations in predicting and preventing AD (Zhu et al., 2006), which means it is one of the possible directions to delay the progression of AD by reducing the impact of risk factors of AD.

Increasing evidence shows that vascular risk factors have emerged as important contributors to AD (Clerici et al., 2012; Rius‐Perez, Tormos, Perez, & Talens‐Visconti, 2018), such as hypertension (Gorelick, 2004; Qiu, 2012), diabetes mellitus (Wijesinghe et al., 2016), cardiovascular (Newman et al., 2005; Stampfer, 2006), and cerebrovascular disease (Snowdon et al., 1997; Yarchoan et al., 2012). Vascular risk factors may even accelerate the progression of AD (Li et al., 2010). Vascular pathology may play an important role in the etiology of AD by decreasing cerebral blood and impairing Aβ clearance (Gupta & Iadecola, 2015). As an important surrogate of vascular pathology (Attems & Jellinger, 2014; Schneider, Arvanitakis, Bang, & Bennett, 2007), atherosclerosis is one of the potential targets to reduce the risk of AD through early screening and intervention (Lopez‐Oloriz et al., 2013; Luzzi, Vella, Bartolini, Provinciali, & Silvestrini, 2010).

However, there are inconsistent associations between atherosclerosis and AD (Beach et al., 2007; Luoto et al., 2009), and there is an absence of suitable measurement to link atherosclerosis and AD. Thus, we performed a systematic review and meta‐analysis of the existing literature to study the association between atherosclerosis and AD.

## 2 METHODS

### 2.1 Search strategy

We manually searched the PubMed, Embase, Cochrane Library, and CNKI (China National Knowledge Infrastructure) to find all articles on Alzheimer's disease and atherosclerosis. The following terms were searched: (Alzheimer Disease OR Alzheimer's disease OR AD) AND (Atherosclerosis OR atherosis OR AS). The search spanned up to September 2018. No language restriction was applied.

### 2.2 Selection criteria

Two investigators independently conducted preliminary searches on the above terms and evaluated the title and abstract after removal of duplicated literature. Then, they, respectively, read 78 full‐text records and assessed the quality of the studies according to the selection criteria, with disagreement resolved by consensus or arbitrated by another investigator. This review was carried out by the Preferred Reporting Items for Systematic Reviews and Meta‐Analyses (PRISMA) statement (Knobloch, Yoon, & Vogt, 2011). The Newcastle–Ottawa Scale (NOS) was used to evaluate article quality. Studies were excluded with the NOS score smaller than 4.

### 2.3 Data extraction and analysis

A single investigator extracted data from the included studies using a designed table, and the other investigator examined the data to ensure accuracy. The obtained data included the first author of the study, the publication time, the study design, sample size in total and in patients, measurement of atherosclerosis and ascertainment of AD.

Eligible trials had to satisfy the following prespecified PICOS criteria: (a) patient: AD patients; (b) intervention: atherosclerosis; (c) comparison: non‐AD population; (d) outcome: the association of atherosclerosis and AD; and (e) study design: case–control study or cohort study. We merely focused on AD though there were many different types of dementia in the trials. We adopted data of multiple measurements of the diagnosis and progression of AD in the qualified studies, including neuroimaging and neuropsychology. In the aspect of atherosclerosis, we extracted data of carotid intima–media thickness (CIMT) and the measurement of the circle of Willis.

### 2.4 Statistical methods

The association between atherosclerosis and AD was measured in two ways. Firstly, we compared CIMT between the AD group and the control group to find whether there is a significant difference in CIMT value between the two groups. We extracted the values of mean CIMT and standard deviation (*SD*) in the relevant studies, and combined the values of standardized mean difference (SMD). Secondly, multiple regression or Cox regression models was adopted to find whether increasing atherosclerosis grade increased the odds ratios (OR) for the diagnoses of AD or not. We extracted the relevant data and calculated the combined OR.

Statistical analysis was carried out by using Revman 5.3, including combined OR and heterogeneity test analysis. A random‐effects model was applied to evaluate the association of atherosclerosis and AD when heterogeneity was found (*p* < .10 or *I* ^2^ > 50%). Funnel plots were used to investigate the publication bias.

The measurement and classification criteria of atherosclerosis and AD were not the same in different studies. However, all the included studies in the review analyzed ORs of atherosclerosis between AD patients and controls from the perspective of their measurement. Thus, the difference in measurement and classification between atherosclerosis and AD among studies can be neglected by combined OR. Concerning mean CIMT, we used combined SMD to ignore the above difference between the AD group and the control group because mean CIMT with different measure and grading standards cannot be directly combined. Data of mean CIMT from different subgroups in one study were premerged to eliminate the difference.

### 2.5 Quality assessment

To assess the quality of nonrandomized studies in the meta‐analysis, we used the Newcastle–Ottawa Quality Assessment Scale (NOQAS), which was applied to evaluate case–control studies and cohort studies. There is a set of eight questions in three major domains covered in the semiquantitative scoring system, including the selection of cases and controls, comparability of selected groups, and exposure or outcome of interest. The score may range from 0 to 8. Two investigators independently assessed the quality of the studies, with disagreement resolved by consensus or arbitrated by another investigator.

### 2.6 Assessment of risk of bias

Funnel plots were used to investigate the publication bias.

## 3 RESULTS

### 3.1 Eligible studies

**Figure 1:**
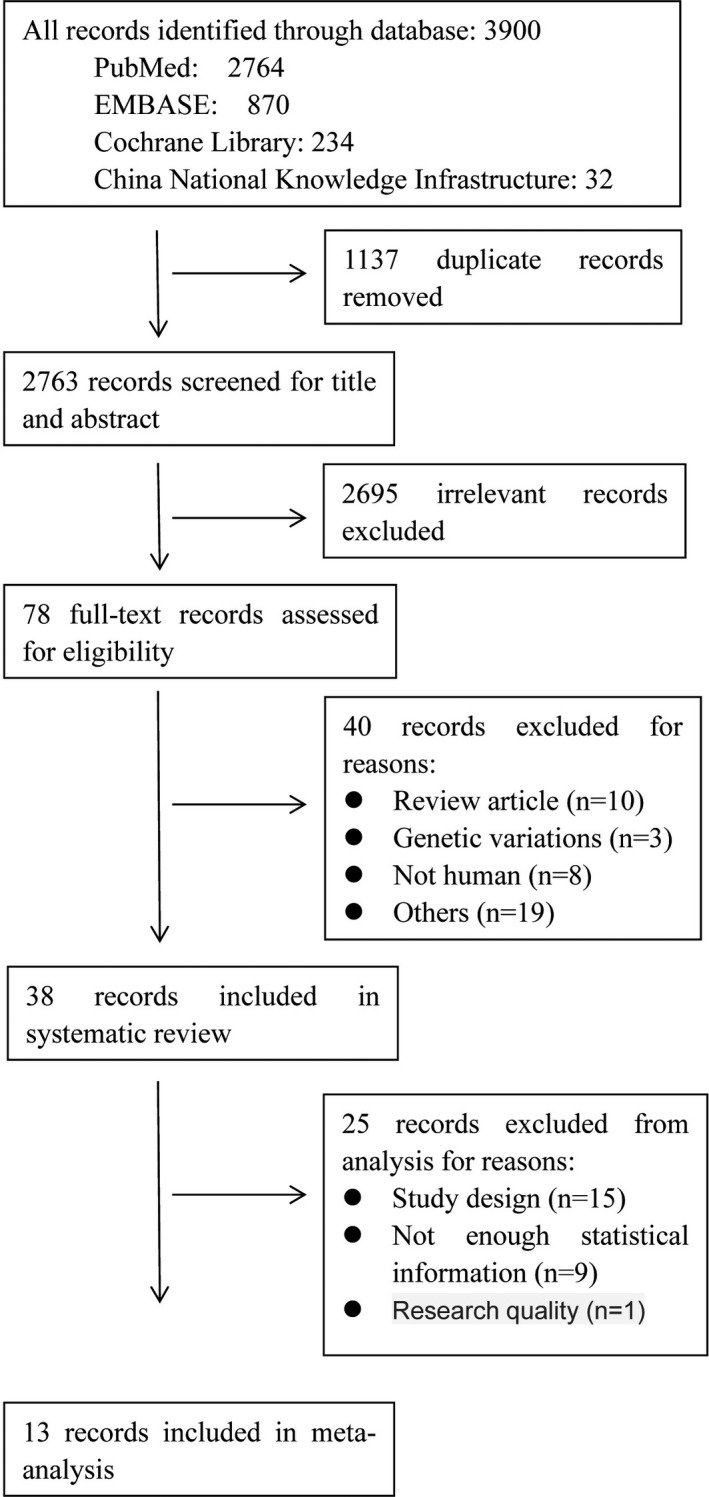
Flowchart of the study selection

The process of article retrieval is shown in Figure 1. A total of 3,900 studies were identified. After the removal of duplicate studies, we screened the titles and abstracts of 2,763 articles and assessed full text of 78 articles for eligibility. Finally, thirteen studies met our inclusion criteria (Figure 1). The main characteristics of the included articles are shown in Table 1.

**Table 1:** Characteristics of included studies

Study name	Study design	Sample size	No AD	Measurement of atherosclerosis	Ascertainment of AD
Arvanitakis et al. (2016)	1	1,142	478	Brain autopsy	MMSE, 17 individual tests of cognition
Beach et al. (2007)	2	398	215	Brain autopsy	NIA/Reagan Institute criteria
Bos et al. (2015)	3	2,364	73	Tomography, quantify atherosclerotic calcification	MMSE, the Stroop test, LDST, WFT, 15‐WLT
Buratti et al. (2015)	3	406	106	BHI	MMSE, the Mental Deterioration Battery, Trail Making A and B, Test of Judgment, Neuropsychiatric Inventory
Hofman et al. (1997)	3	1982	207	CIMT	DSM‐III‐R criteria, NINCDS‐ADRDA
Li, Zhou and Wang (2016)	2	113	53	CIMT	NINCDS‐ADRDA
Qiu et al. (2010)	2	1,270	328	Vascular risk factors or vascular disorders	DSM‐III‐R criteria
Roher et al. (2011)	2	97	61	Brain autopsy	MMSE
Tan et al. (2009)	2	175	64	CIMT	MMSE
Xiao et al. (2011)	2	186	106	CIMT	NINCDS‐ADRDA
Yue, Liu and Tang (2011)	2	51	21	CIMT	ICD ‐10 (1992)
Zhang & Gao (2015)	2	272	136	CIMT	NINCDS‐ADRDA
Zhou et al. (2016)	2	130	60	CIMT	MMSE

Note

Study design: 1. Cross‐sectional study; 2. case–control study; 3. prospective cohort study.

Abbreviations: 15‐WLT, 15‐word verbal learning task; AD, Alzheimer's disease; BHI, breath‐holding index; CIMT, carotid intima–media thickness; DSM‐III‐R, the Diagnostic and Statistical Manual of Mental Disorders, Third Edition, Revised criteria; ICD‐10, the International Statistical Classification of Diseases and Related Health Problems 10th Revision; LDST, the Letter‐Digit Substitution Task; MMSE, Mini‐Mental State Examination; NIA/Reagan Institute criteria, the National Institute on Aging and the Reagan Institute Working Group on diagnostic criteria for the neuropathological assessment of Alzheimer disease; NINCDS‐ADRDA, criteria of the working group of the National Institute of Neurological and Communicative Disorders and Stroke and the Alzheimer's Disease and Related Disorders Association; WFT, word fluency test.

### 3.2 Description of studies

The 13 studies identified for analysis were published between 1997 and 2016 (Table 1). Of the 13 studies (Arvanitakis, Capuano, Leurgans, Bennett, & Schneider, 2016; Beach et al., 2007; Bos et al., 2015; Buratti et al., 2015; Hofman et al., 1997; Li, Zhou, & Wang, 2016; Qiu, Xu, Winblad, & Fratiglioni, 2010; Roher et al., 2011; Xiao, Yi, Zhou, & Feng, 2011; Tan, Ma, Liu, & Shi, 2009; Yue, Liu, & Tang 2011; Zhang Zhihua, 2015; Zhou, He, Wang, Ding, & Lu, 2016), 10 were case–control studies (Arvanitakis et al., 2016; Beach et al., 2007; Li, Zhou, & Wang, 2016; Qiu et al., 2010; Roher et al., 2011; Xiao et al., 2011; Tan et al., 2009; Yue Xiaobin & Weidong, 2011; Zhang Zhihua, 2015; Zhou et al., 2016), and three were prospective cohort study (Bos et al., 2015; Buratti et al., 2015; Hofman et al., 1997). Seven of the 13 studies provided data for the mean CIMT in subjects with and without AD, and ten studies used a logistic regression model to analyze the prevalence of atherosclerosis in subjects with and without AD. Four studies were adopted to analyze both aspects. In the course of analysis, we focused on the evaluation of atherosclerosis and the ascertainment of AD.

### 3.3 Meta‐synthesis of results

To assess the relationship between atherosclerosis and AD, we explored the differences in the severity and the prevalence of atherosclerosis between AD and control groups.

#### 3.3.1 Standard mean difference of CIMT

**Figure 2:**
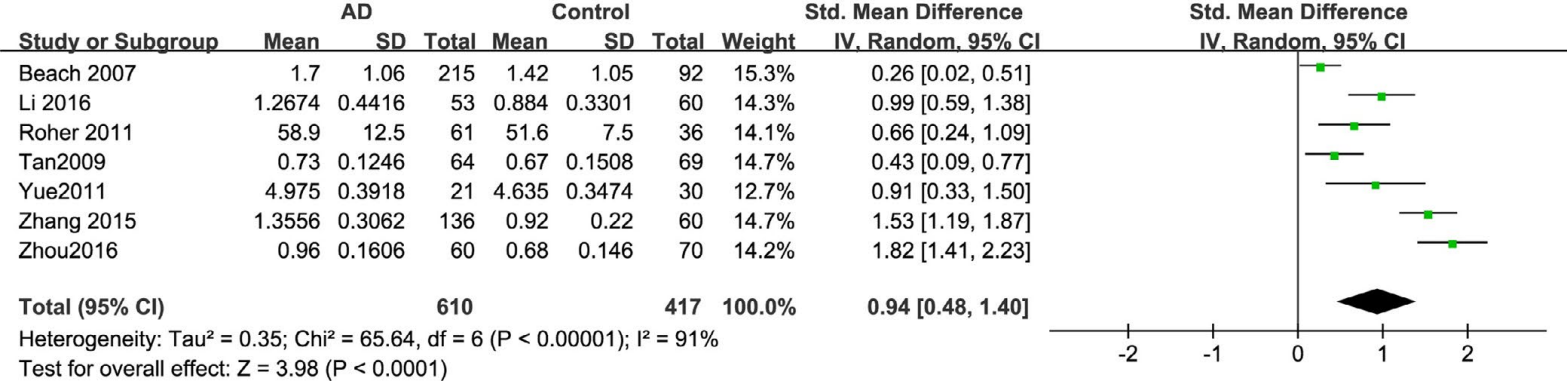
Forest plot showing the standard mean difference (SMD) of carotid intima–media thickness (CIMT) between AD patients and controls in a random‐effects model

Seven studies assessed the CIMT difference between AD patients and controls (Beach et al., 2007; Li et al., 2016; Roher et al., 2011; Tan et al., 2009; Yue Xiaobin & Weidong, 2011; Zhang Zhihua, 2015; Zhou Rujuan et al., 2016). As shown in Figure 2, a total of 1,027 subjects were included in the analysis, 610 subjects with AD patients and 417 subjects without AD. We premerged data of mean value in subgroups and found that *p*‐value in each included study was <.05, which demonstrated a significant difference of CIMT between the AD group and the control group. Then, we used an inverse variance model to combine the mean value. Patients with AD had a higher mean CIMT than subjects without AD (overall SMD = 0.94; 95% CI, 0.48–1.40; *p* < .0001; *I* ^2^ = 91%). Due to large heterogeneity, a random‐effects model was used to combine the data. The result showed that there was a significant statistically difference of CIMT between patients with and without AD.

#### 3.3.2 Odds ratio of atherosclerosis

**Figure 3:**
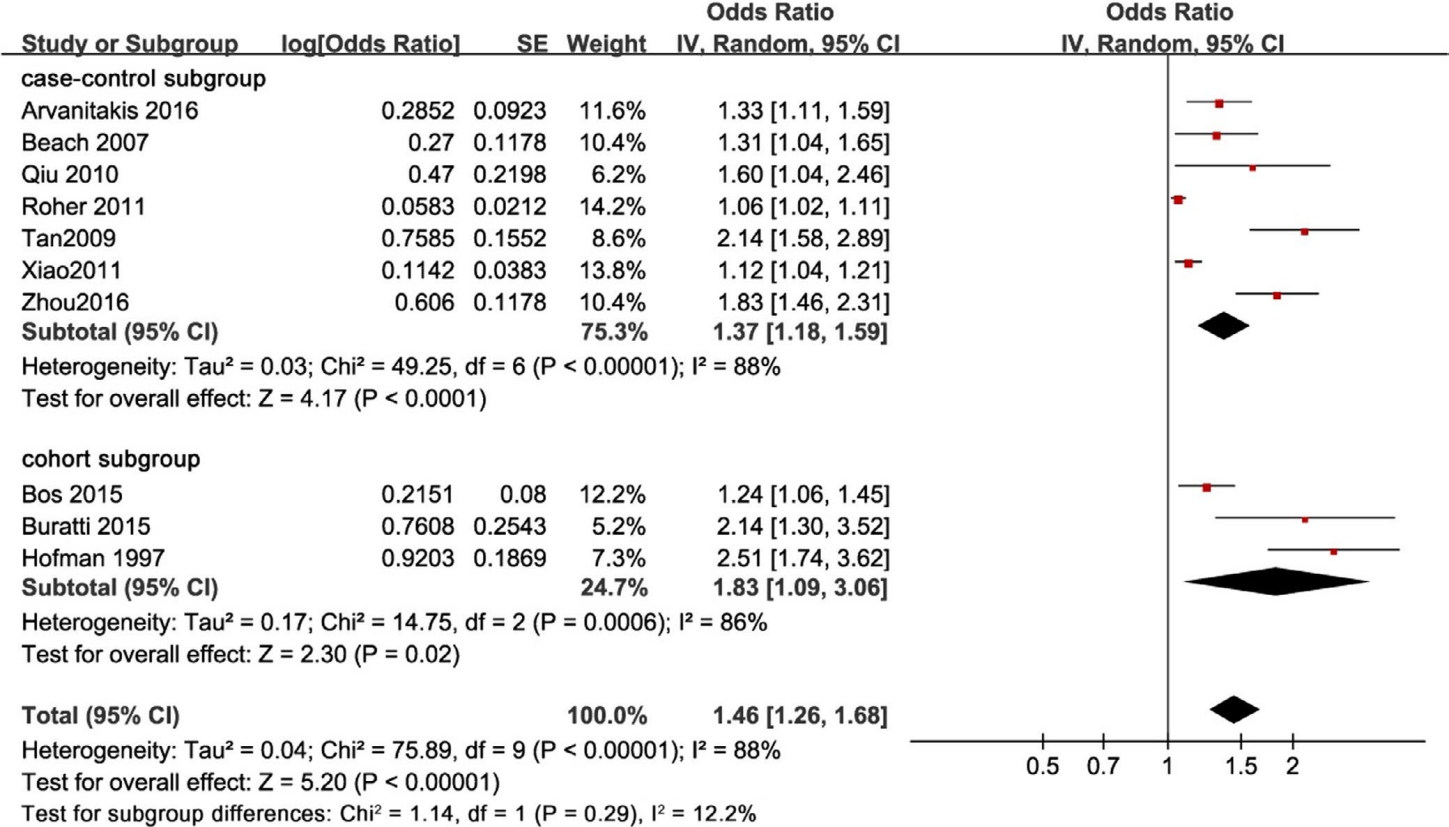
Forest plot showing the combined odds ratio (OR) for the risk of Alzheimer's disease (AD) in patients

Ten studies were analyzed to evaluate the prevalence of atherosclerosis between subjects with and without AD (Arvanitakis et al., 2016; Beach et al., 2007; Bos et al., 2015; Buratti et al., 2015; Hofman et al., 1997; Qiu et al., 2010; Roher et al., 2011; Tan et al., 2009; Xiao et al., 2011; Zhou et al., 2016). Before the analysis, data of the OR value in subgroups in two studies were preprocessed into the combined OR (Hofman et al., 1997; Qiu et al., 2010) and then the OR values of these ten studies were combined. Based on the study design of these 10 studies, we divided them into two subgroups to separately calculate the OR of atherosclerosis (Figure 3). In the case–control subgroup (Arvanitakis et al., 2016; Beach et al., 2007; Qiu et al., 2010; Roher et al., 2011; Tan et al., 2009; Xiao et al., 2011; Zhou et al., 2016), the OR of atherosclerosis is 1.37 (95% CI, 1.18–1.59; *p* < .0001; *I* ^2^ = 88%). In the cohort subgroup (Bos et al., 2015; Buratti et al., 2015; Hofman et al., 1997), the OR of atherosclerosis is 1.83 (95% CI, 1.09–3.06; *p* < .05; *I* ^2^ = 86%). All the evaluated studies indicated that atherosclerosis is related to a higher risk of AD after adjustment for relevant confounders, with an OR in each study >1 and *p*‐value of each study <.05. The prevalence of atherosclerosis was significantly higher in subjects with AD compared to those without, with an overall OR with an inverse variance model of 1.46 and 95% CI of 1.26–1.68 (Figure 3). It is important to point out that we extracted risk estimates for the most adjusted models. There was significant heterogeneity between the studies (*I* ^2^ = 88%, *p* < .00001). Thus, we used a random‐effects model to combine the data. The result showed that atherosclerosis is associated with a high risk of AD.

### 3.4 Publication bias and study quality

The quality of studies, as assessed by NOS, is summarized in Table 2. The NOS scores of all the included studies are at least 4 points.

**Table 2:** The NOS for assessing the quality of studies

Reference	Selection	Comparability	Exposure/outcome	Overall NOS scores
Arvanitakis et al. (2016)	☆☆☆☆	☆	–	5
Beach et al. (2007)	☆☆☆☆	☆	☆☆	7
Bos et al. (2015)	☆☆☆	☆☆	☆☆☆	8
Buratti et al. (2015)	☆☆☆	☆☆	☆☆☆	8
Hofman et al. (1997)	☆☆☆	☆	☆☆☆	7
Li et al. (2016)	☆☆☆	☆	☆	5
Qiu et al. (2010)	☆☆☆☆	☆☆	☆☆	8
Roher et al. (2011)	☆☆☆☆	☆	☆☆	7
Tan et al. (2009)	☆☆☆	☆☆	☆	6
Xiao et al. (2011)	☆☆☆	☆	☆☆	6
Yue Xiaobin & Weidong (2011)	☆☆☆	–	☆	4
Zhang Zhihua (2015)	☆☆☆	☆☆	☆	6
Zhou et al. (2016)	☆☆☆	☆	–	4

### 3.5 Assessment of quality of the included studies

**Figure 4:**
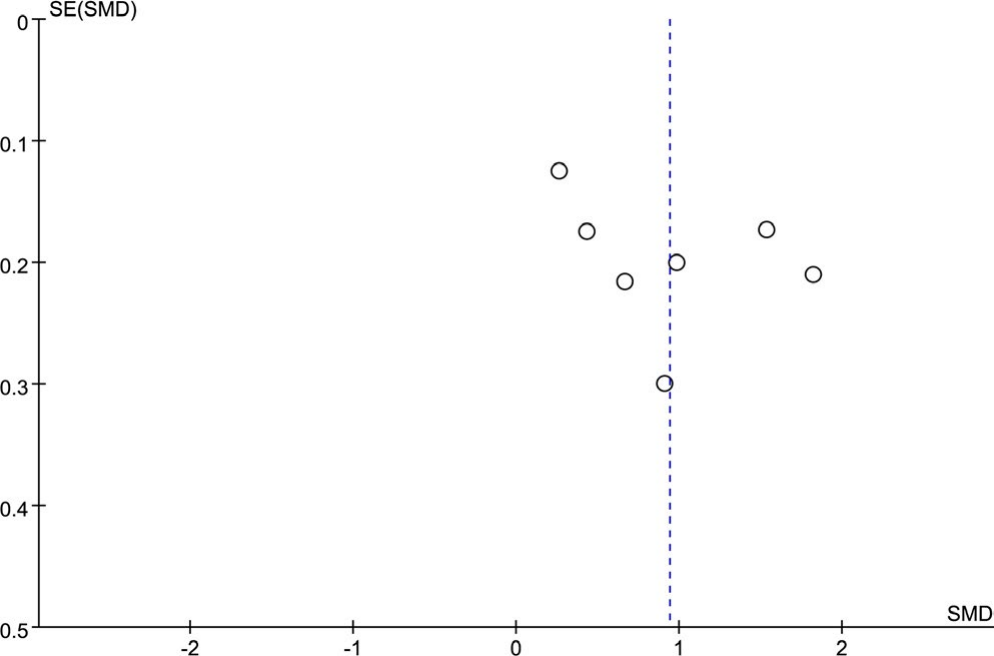
Funnel plot of the standard mean difference (SMD) of carotid intima–media thickness (CIMT) between AD patients and controls showing no publication bias in visual

**Figure 5:**
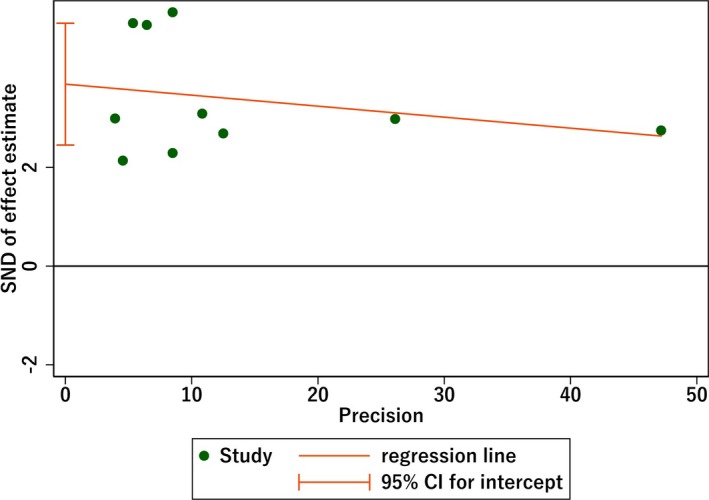
Funnel plot of the odds ratio (OR) for patients with atherosclerosis compared with controls showing publication bias

**Figure 6:**
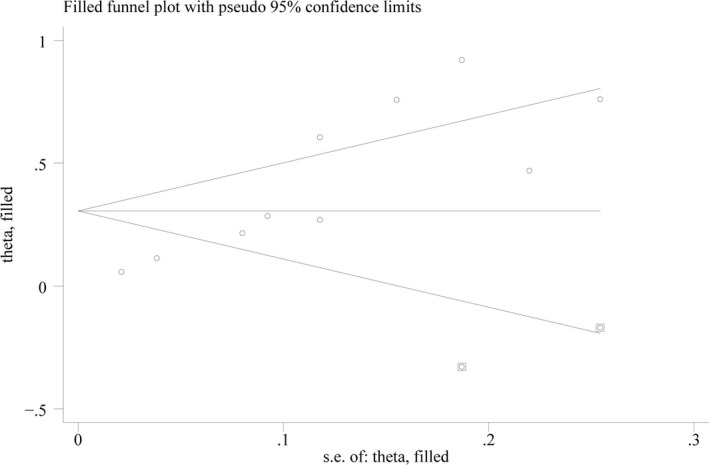
Filled funnel plot for the association between atherosclerosis and AD showing an unbiased state by filling two studies

Funnel plots were used to assess the publication bias across the studies (Figures 4, 5 and 6). The shape of the funnel plot generated in the analysis of the CIMT difference between patients and controls was symmetrical (Figure 4). When it comes to the OR for patients with atherosclerosis compared with controls, the Egger's test indicated publication bias (*p* < .001; Figure 5). Thus, we conducted a trim‐and‐fill analysis for this comparison (Figure 6), which showed an unbiased state by filling two studies. However, no proper research has been searched to fill the analysis.

### 3.6 Additional analysis

**Figure 7:**
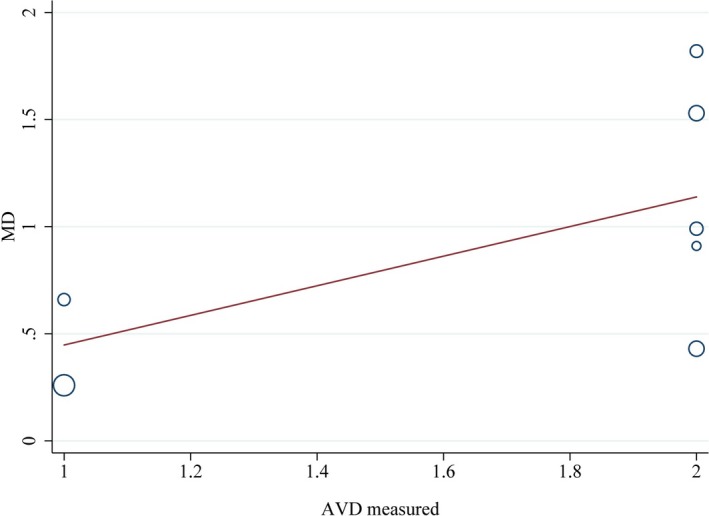
Meta‐regression of the standard mean difference (SMD) of carotid intima–media thickness (CIMT) between AD patients and controls—atherosclerosis vascular disease (AVD) measured method

**Figure 8:**
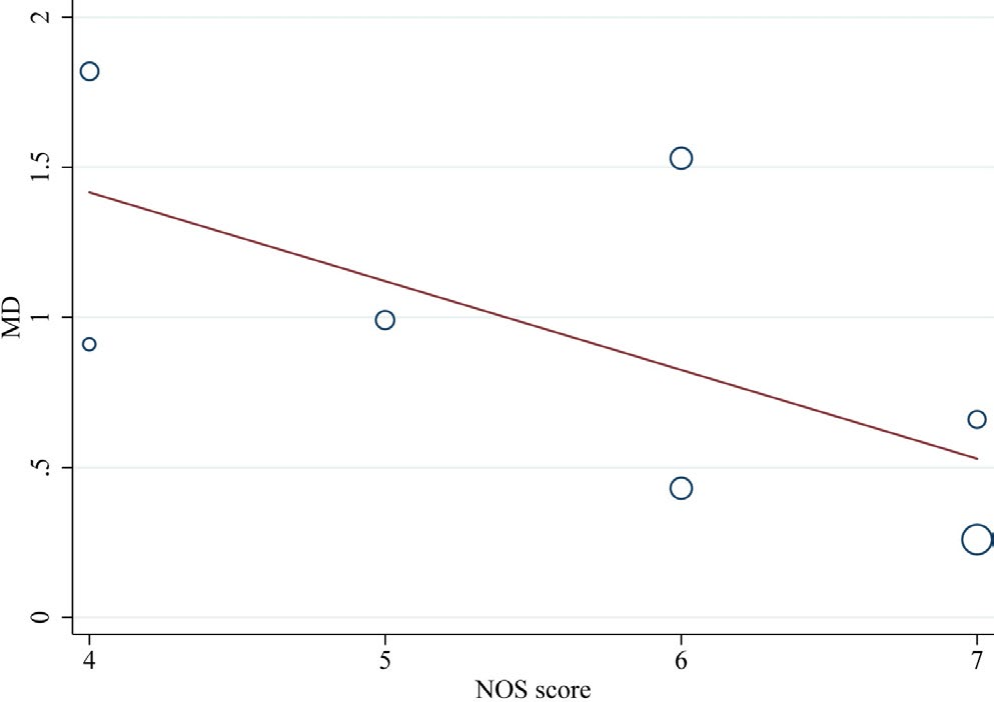
Meta‐regression of the standard mean difference (SMD) of carotid intima–media thickness (CIMT) between AD patients and controls‐NOS score

**Figure 9:**
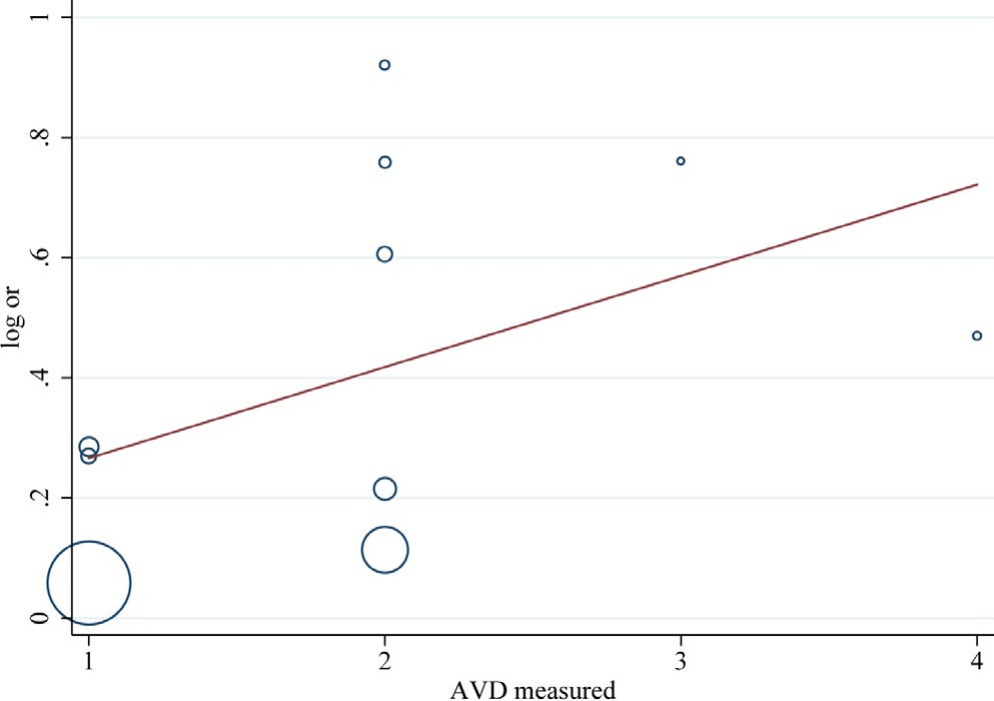
Meta‐regression of the odds ratio for the risk of AD—atherosclerosis vascular disease (AVD) measured method

Leave‐one‐out study analysis was conducted as a component of sensitivity analysis showing that the findings were not driven by any single study. We made meta‐regression analyses to explore whether these five covariates (sample size, country, atherosclerotic vascular disease [AVD] measured method, ascertainment of AD, and NOS score) were the source of heterogeneity. The results showed that AVD measured method contributed 24.9% to heterogeneity of the combined effect value of SMD of CIMT with the chi‐square value decreasing from 94% to 86% (Figure 7). NOS score had 34.9% contribution to heterogeneity of the combined effect value of SMD of CIMT resulting in the chi‐square value dropping to 84.6% from 94% (Figure 8). AVD measured method contributed 15% to heterogeneity of the combined effect value of OR of atherosclerosis, which declined from 88% to 85.6% (Figure 9). No other covariates were found to contribute to heterogeneity.

## 4 DISCUSSION

The purpose of this systematic review and meta‐analysis is to investigate the relationship between atherosclerosis and AD. We included 13 studies with a total of 8,586 samples. Ten of them are case–control studies, and three are prospective cohort studies. We analyzed the relationship between atherosclerosis and AD from two perspectives. Our meta‐analysis found that the CIMT in the AD group is significantly higher than that in the control group, and the prevalence of atherosclerosis is higher in patients with AD than those without AD. The data suggested that atherosclerosis is significantly associated with AD.

So far, no agreement has been reached on the relationship between atherosclerosis and AD. A growing body of evidence showed that there is an association between atherosclerosis and AD (Beach et al., 2007; Roher et al., 2003, 2011; Roher, Esh, Rahman, Kokjohn, & Beach, 2004). Epidemiological studies have shown that both of them share many vascular risk factors, such as age, obesity, smoking, hypertension, diabetes mellitus, hypercholesterolemia, hyperhomocysteinemia, and APOE4 isoforms (Gorelick et al., 2011). Many studies have explored the relationship between atherosclerosis and AD. In a case–control study, Roher et al. measured a total of 2,108 cross sections of the blood vessels of the Circle of Willis, revealing that the degree of stenosis of major intracranial blood vessels was higher in patients with AD than in the control group (Roher et al., 2011). A study with 130 samples suggested a negative correlation between CIMT and the MMSE score (Zhou et al., 2016). On the other hand, some researchers found no significant association between the severity of stenosis of carotid and intracranial arteries and cognitive impairment (Lopez‐Oloriz et al., 2013), and decreased brain blood flow caused by atherosclerosis might not lead to increased deposition of Aβ and hyperphosphorylation of tau protein (Hansson et al., 2018).

Based on the unclear association between atherosclerosis and AD, we performed this systematic review and meta‐analysis. Our meta‐analysis showed that the prevalence of atherosclerosis is higher in the AD group than in the control group. Currently, there is a lack of appropriate markers to evaluate the role of atherosclerosis and other vascular risk factors in the process of AD. In clinical practice, CIMT is a widely used measure for early atherosclerosis, and it can be simply and noninvasively measured by B‐mode ultrasound (Oygarden, 2017). So, we analyzed the difference of CIMT between the AD group and the control group. Our data showed that CIMT is significantly increased in the AD group compared to the control group. Thus, CIMT might be a useful marker to reflect the risk of cognitive decline (Lorenz, Markus, Bots, Rosvall, & Sitzer, 2007; Urbanova et al., 2014), in addition to assessing vascular burden.

Many studies suggested that atherosclerosis plays an important role in the process of occurrence and development of AD. Atherosclerosis can lead to neuronal damage and cognitive decline through a variety of pathways. Hypoperfusion and hypoxia in the brain caused by atherosclerosis accelerated the overproduction of Aβ both in models of AD and in individuals with AD (Garcia‐Alloza et al., 2011; Iadecola, 2004; Koike, Green, Blurton‐Jones, & Laferla, 2010; Park et al., 2008). In a low‐oxygen environment, the cleavage of Aβ peptides from amyloid‐β protein precursor (AβPP) significantly increases via overexpression of β‐ and γ‐secretases and downregulation of α‐secretase, which leads to an increase in the production of Aβ (Li et al., 2009; Salminen, Kauppinen, & Kaarniranta, 2017; Sun et al., 2006; Zhang et al., 2007). In turn, the overproduction of Aβ has an adverse effect on cerebrovascular function (Iadecola, 2004; Thomas, Thomas, McLendon, Sutton, & Mullan, 1996), such as reduced blood‐vessel compliance, which could influence brain blood flow and thus form a vicious cycle. On the other hand, atherosclerosis reduces Aβ clearance in the brain through affecting the transport across the blood–brain barrier (BBB; Yamazaki & Kanekiyo, 2017), the perivascular pathway, and the glymphatic pathway, as well as enzymatic degradation (Bell & Zlokovic, 2009). The disruption of the neurovascular unit resulted by atherosclerosis might also contribute to oxidative stress and nitrosative damage (Chaitanya et al., 2012; Girouard & Iadecola, 2006), which may lead to metabolic poison generation and neuroinflammatory response, and vice versa. Decreased Aβ clearance combined with the overproduction of Aβ and oxidative stress accelerates Aβ deposition, which may ultimately lead to cognitive impairment (Chetelat et al., 2012; Dore et al., 2013).

Atherosclerosis is associated with many vascular factors, such as hypertension, diabetes mellitus, dyslipidemia, and smoking. The management of these risk factors can prevent and modify the incidence and progress of atherosclerosis, which may play a crucial role in delaying the process of AD (Luzzi et al., 2010; Perlmutter, 2016). A study on the influence of risk factor control on the prevalence of AD estimated that at least 640,000 AD cases could be expected to be prevented worldwide if there were a 10% reduction separately in the prevalence of hypertension, diabetes, and smoking (Barnes & Yaffe, 2011). Other studies found that the drug use of statins, a class of cholesterol‐lowering drugs, was associated with decreased risk of AD (Geifman, Brinton, Kennedy, Schneider, & Butte, 2017; Zhang, Wen, & Zhang, 2018). Our data provided further evidence that atherosclerosis is significantly associated with AD, which supports the view that relieving atherosclerosis may probably reduce the risk of AD.

Some limitations should be considered. First, the diagnostic criteria for AD in the included studies were inconsistent, half of which was based on clinical data records and the rest on the MMSE score. Second, we did not pre‐establish exclusion rules about the different stages of AD, which means patients were included in all stages of AD. Third, because of the heterogeneity of the study, we used a random‐effects model to combine effect values to reduce the effects of heterogeneity. We did not find the source of heterogeneity through the meta‐regression.

## 5 CONCLUSIONS

In summary, our systematic review and meta‐analysis show that atherosclerosis is strongly associated with AD, which may provide potential strategies for prevention and therapy of AD. In the future, more studies need to be done on whether different degrees of atherosclerosis are associated with different levels of cognitive decline in AD and on whether CIMT is a potential biomarker to predict cognitive decline.

## CONFLICT OF INTEREST

The authors declare that the research was conducted in the absence of any commercial or financial relationships that could be construed as a potential conflict of interest.

## AUTHOR'S CONTRIBUTION

Y.T. and B.X. conceived the study and wrote the paper; B.X. and X.S. performed the literature search, data extraction, and the statistical analysis; and Y.X. and Y.T. reviewed the manuscript.

## Data Availability

The data that support the findings of this study are available from the corresponding author, Y.T., upon reasonable request.
